# Prehospital blood pressure lowering in acute hemorrhagic stroke: a systematic review and meta-analysis of randomized controlled clinical trials

**DOI:** 10.1093/esj/aakaf023

**Published:** 2026-01-01

**Authors:** Aikaterini Theodorou, Konstantinos Melanis, Lina Palaiodimou, Georgia Papagiannopoulou, Eleni Bakola, Maria Chondrogianni, Apostolos Safouris, Alexandra Frogoudaki, Ioanna Koutroulou, Theodoros Karapanayiotides, Effrosyni Koutsouraki, Silke Walter, Maren Ranhoff Hov, Janika Kõrv, Else Charlotte Sandset, Efstathios Manios, Georgios Tsivgoulis

**Affiliations:** Second Department of Neurology, School of Medicine, National and Kapodistrian University of Athens, “Attikon” University Hospital, Athens, Greece; Second Department of Neurology, School of Medicine, National and Kapodistrian University of Athens, “Attikon” University Hospital, Athens, Greece; Second Department of Neurology, School of Medicine, National and Kapodistrian University of Athens, “Attikon” University Hospital, Athens, Greece; Second Department of Neurology, School of Medicine, National and Kapodistrian University of Athens, “Attikon” University Hospital, Athens, Greece; Second Department of Neurology, School of Medicine, National and Kapodistrian University of Athens, “Attikon” University Hospital, Athens, Greece; Second Department of Neurology, School of Medicine, National and Kapodistrian University of Athens, “Attikon” University Hospital, Athens, Greece; Second Department of Neurology, School of Medicine, National and Kapodistrian University of Athens, “Attikon” University Hospital, Athens, Greece; Stroke Unit, Metropolitan Hospital, Piraeus, Greece; Second Department of Cardiology, School of Medicine, National and Kapodistrian University of Athens, “Attikon” University Hospital, Athens, Greece; Second Department of Neurology, School of Medicine, Aristotle University of Thessaloniki, AHEPA University Hospital, Thessaloniki, Greece; Second Department of Neurology, School of Medicine, Aristotle University of Thessaloniki, AHEPA University Hospital, Thessaloniki, Greece; First Department of Neurology, School of Medicine, Aristotle University of Thessaloniki, AHEPA University Hospital, Thessaloniki, Greece; Neurology, Saarland University, Homburg, Germany; Department of Research, Norwegian Air Ambulance Foundation, Oslo, Norway; Stroke Unit, Department of Neurology, Oslo University Hospital, Oslo, Norway; Bachelor of Prehospital Medicine, Oslo Metropolitan University, Oslo, Norway; Department of Neurology and Neurosurgery, University of Tartu, Tartu, Estonia; Department of Research, Norwegian Air Ambulance Foundation, Oslo, Norway; Stroke Unit, Department of Neurology, Oslo University Hospital, Oslo, Norway; Department of Clinical Therapeutics, “Alexandra” General Hospital, School of Medicine, National and Kapodistrian University of Athens, Athens, Greece; Second Department of Neurology, School of Medicine, National and Kapodistrian University of Athens, “Attikon” University Hospital, Athens, Greece; Department of Neurology, University of Tennessee Health Science Center, Memphis, TN, United States

**Keywords:** blood pressure, hemorrhagic stroke, meta-analysis, prehospital management

## Abstract

**Introduction:**

Elevated blood pressure (BP) in acute hemorrhagic stroke has been associated with adverse clinical outcomes. Limited data from randomized controlled clinical trials (RCTs) indicate that early BP management, in the prehospital setting, may be safe and beneficial. We sought to evaluate the efficacy and safety of prehospital BP-lowering in acute hemorrhagic stroke when compared to usual care.

**Patients and methods:**

We conducted a systematic review and meta-analysis including available RCTs evaluating prehospital BP-lowering among acute hemorrhagic stroke patients. The pooled risk ratio (RR) of a 3-month good functional outcome, defined as modified-Rankin-Scale scores of 0-2 and all-cause 3-month mortality were the primary efficacy and safety outcomes, respectively. Secondary outcomes included the pooled RR of hematoma expansion (HE) and serious adverse events (SAEs).

**Results:**

A total of four RCTs were included, comprising 642 patients treated with prehospital BP-lowering therapies and 617 patients receiving usual care. Prehospital BP-lowering was associated with similar rates of good functional outcome (RR: 1.07; 95% CI, 0.52–2.19) and all-cause mortality (RR: 0.90; 95% CI, 0.60–1.35) at 3 months, compared to usual care. The risk of SAEs (RR: 0.97; 95% CI, 0.74–1.26) and HE (RR: 1.05; 95% CI, 0.45–2.46) did not significantly differ between the two groups. Subgroup analyses revealed the superiority of the α-adrenoreceptor blocker urapidil compared to glyceryl trinitrate in terms of reducing SAE risk and HE.

**Conclusion:**

Our meta-analysis indicates that prehospital BP-lowering in acute hemorrhagic stroke does not improve functional outcome and survival. Future RCTs conducted in mobile stroke units, and exclusively focusing on patients with acute hemorrhagic stroke, are required.

## Graphical Abstract

**Graphical abstract:**
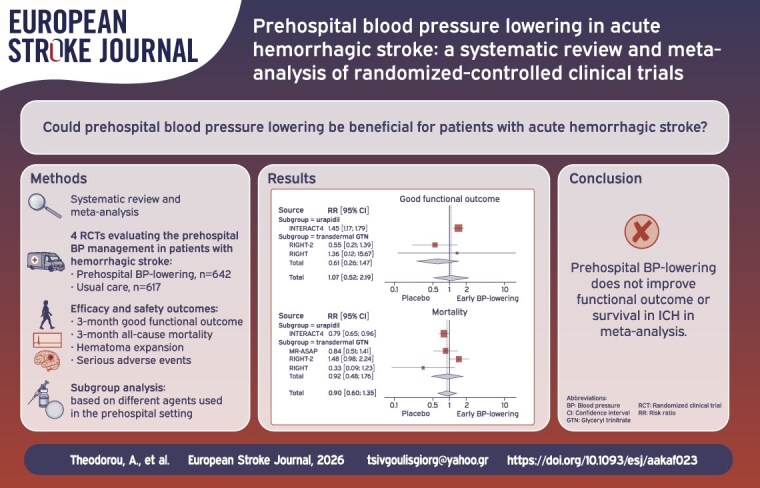

## Introduction

Elevated blood pressure (BP) is common in acute intracerebral haemorrhage (ICH) and has been associated with a higher risk of hematoma expansion (HE), unfavourable functional outcomes and higher mortality rates.^1–4^ Early treatment initiation for BP lowering is recommended by current international recommendations.^5^^,^^6^ Nevertheless, the optimal strategy for BP management in patients with acute ICH and the safety and efficacy of BP lowering in the prehospital setting remain a subject of ongoing investigation and debate.

Recent randomized controlled clinical trials (RCTs) have investigated prehospital BP management among patients with suspected, however undifferentiated stroke in the prehospital setting.^7–11^ These studies failed to demonstrate significant benefit of prehospital BP management for acute ischemic stroke, probably attributed to the compromised cerebral blood flow in the evolving ischemic penumbra caused by early blood-pressure reduction.^10^ Notably, potential harm in patients with ICH were documented in RIGHT-2 and MR ASAP, leading to premature termination of MR ASAP due to safety concerns of the intervention.^9^ However, the recently published INTERACT4 showed lower odds of poor functional outcome among patients with acute hemorrhagic stroke, receiving prehospital BP lowering intervention compared to usual care.^10^ Nevertheless, no RCT evaluated prehospital BP management among acute ICH patients diagnosed in the prehospital setting using mobile stroke units (MSUs).

In view of the former considerations, we sought to evaluate the efficacy and safety of prehospital BP management compared to usual care among patients with acute hemorrhagic stroke. We also assessed the potential effect of different antihypertensive agents, administered in the prehospital setting on predefined outcomes.

## Patients and methods

### Data availability

All data analyzed in this study are included in the current manuscript and its online supplemental information files.

### Standard protocol approvals, registrations and patient consents

The prespecified protocol for this systematic review and meta-analysis has been registered in the International Prospective Register of Ongoing Systematic Reviews PROSPERO database (Registration ID: CRD420251067577; date of registration: 4 June 2025) and adheres to the updated Preferred Reporting Items for Systematic Reviews and Meta-Analyses (PRISMA) guidelines.^12^ Ethical board approval or written informed consent from patients was not necessary because of the study’s design (systematic review and meta-analysis).

### Data sources and database searches

A systematic literature search was conducted according to the patient, intervention, comparison and outcome model to identify eligible RCTs, including adult patients (≥18 years old) with acute hemorrhagic stroke and elevated systolic BP (P: population) who received treatment to lower the systolic BP at a prehospital setting (I: intervention) vs usual care, which included either placebo at the prehospital setting or usual BP management on arrival at the hospital (C: comparator).^13^ Reporting of any of the outcomes of interest as outlined below (O: outcome) was required for the studies to be considered eligible for inclusion in this meta-analysis.

In accordance with the INTERACT4 trial design—the only trial to include both ICH and a small group (12 patients) with subarachnoid haemorrhage under the collective term “hemorrhagic stroke”—this broader terminology was preferred over “ICH”.^10^

The literature search was conducted independently by 4 reviewers (A.T., K.M., L.P. and G.P.). We searched PUBMED and Scopus, using search strings that included the following terms: “prehospital”, “intracerebral haemorrhage”, “hypertension”, “management”, “functional outcome”, “mortality”, “outcomes”; the complete search algorithms used in PUBMED and Scopus are provided in the Supplement. No language or other restrictions were applied. The search spanned from each electronic database’s inception to 8 June 2025. An additional manual search of bibliographies of articles meeting study inclusion criteria was conducted to ensure the comprehensiveness of the literature. All retrieved studies were independently assessed by 4 reviewers (A.T., K.M., G.P. and L.P.), and any disagreements were resolved after discussion with a fifth tie-breaking evaluator (G.T.).

Exclusion criteria comprised: (1) studies that did not report data with regard to prehospital management of elevated BP among patients with acute hemorrhagic stroke; (2) studies with overlapping data; (3) observational cohort studies, non-controlled studies, case series and case reports; (4) reported outcomes not aligned with our inclusion criteria and (5) narrative and systematic reviews, commentaries, pre-prints or non-paper reviewed studies and conference abstracts.

### Outcomes

The primary efficacy outcome of interest was good functional outcome at 3 months, defined as modified Rankin Scale (mRS) scores of 0-2. The primary safety outcome was all-cause mortality at 3 months.

Secondary outcomes included HE [defined as either absolute (≥6 mL) or proportional (≥33%) hematoma growth at 24 h] and any serious adverse event (SAE, defined by each individual trial, to include those fatal, non-fatal and treatment related adverse events).

### Data extraction

All retrieved studies were independently assessed by 4 reviewers (A.T., K.M., E.B. and M.C.), and any disagreements were resolved after discussion with a fifth tie-breaking evaluator (G.T.). The following information was extracted in structured forms: trial names, first author and year of publication, study design and period of enrollment, total number of study participants, patients’ characteristics and outcomes of interest, including 3-month mRS score of 0-2, 3-month mortality, any reported SAEs and HE, and hypertensive agents administered during the prehospital management.

### Quality control and bias assessment

Quality control and bias assessment among eligible studies were performed, employing the Cochrane Collaboration tool (RoB V.2) for RCTs, independently by 4 authors (A.T., K.M., L.P. and G.P.).^14^ Any disagreements were settled by consensus after discussion with the corresponding author (G.T.).

### Statistical analysis

Meta-analysis was performed using the R-software version 2025.05.0+496 (packages: meta and metafor).^15^ For the pairwise meta-analysis, we calculated for each dichotomous outcome of interest the corresponding risk ratios (RRs) with 95% CI for the comparison of outcome events among patients receiving early BP treatment at prehospital setting vs usual care. A pre-specified subgroup analysis was conducted stratified by the antihypertensive agent used (urapidil; transdermal glyceryl trinitrate). The random-effects model was used to calculate the pooled estimates.^16^

The threshold for statistical significance for the above analyses was set at a two-sided *P* value of <.05. Heterogeneity between studies was assessed with the Cochran *Q* and *I*^2^ statistics. A *P*-value, resulting from the Cochran’s *Q* test and <0.1 indicated heterogeneity. Moreover, and for the qualitative interpretation of the heterogeneity, *I*^2^ values <25%, between 25% and 50% and *I*^2^ > 50% indicated low, moderate and significant heterogeneity, respectively. Small-study effects, as a proxy for publication bias, were assessed when at least four studies were included in the analysis of the outcomes of interest, using both funnel plot inspection and the Egger’s linear regression test.^17^

## Results

### Literature search and included studies

**Figure 1:**
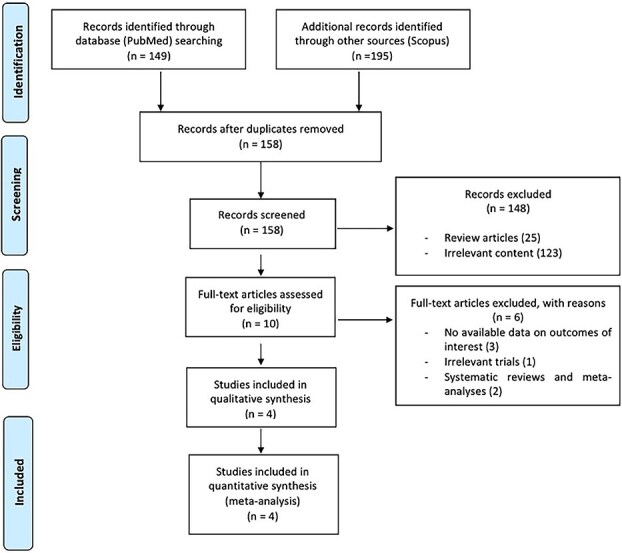
PRISMA flowchart diagram presenting the selection of eligible studies. Abbreviation: PRISMA = Preferred Reporting Items for Systematic Reviews and Meta-Analyses.

The flow diagram for the selection and inclusion of RCTs in this systematic review and meta-analysis is presented in Figure 1. After excluding duplicates, the systematic literature search yielded a total of 158 records. Following the initial screening process, the full texts of 10 studies were retrieved. After evaluating the full-text articles, 6 studies were further excluded due to non-available of data on outcomes of interest, irrelevant content and study design (systematic review) (Table S1). Finally, 4 eligible studies (Table 1) were included in the systematic review and meta-analysis, comprising a total of 642 acute hemorrhagic stroke patients treated with prehospital BP-management (mean age 73.5, 53% men) vs 617 patients with acute hemorrhagic stroke (mean age 72.7, 71 % men), receiving usual care.^7–10^ Radiological diagnosis of acute hemorrhagic stroke was established upon hospital admission in all studies.

### Quality control of included studies

The risk of bias in the included cohort studies was assessed by the ROB-2 tool and is presented in the Figures S1 and S2. The risk of bias in the included studies was low, with only some concerns related to the randomization process followed in the RIGHT study.^7^

### Quantitative analyses

The baseline characteristics of the included studies are presented in Table 1. Moreover, an overview of analyses for primary and secondary outcomes is summarized in Table 2.

**Figure 2:**
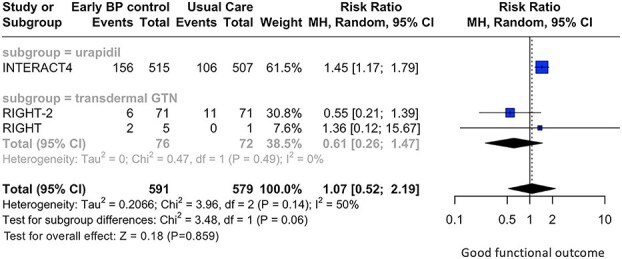
Forest plot presenting the risk ratio of good functional outcome at 3 months among patients with acute hemorrhagic stroke receiving prehospital blood pressure management vs usual care, stratified by the antihypertensive agent administered. Abbreviation: GTN = glyceryl trinitrate.

**Figure 3:**
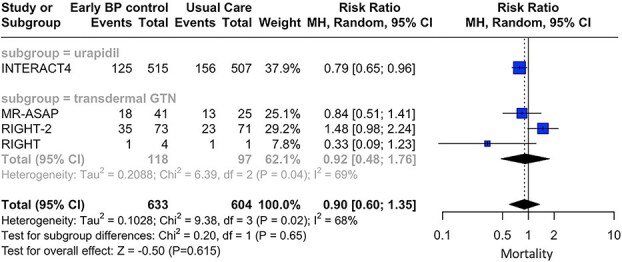
Forest plot presenting the risk ratio of mortality at 3 months among patients with acute hemorrhagic stroke receiving prehospital blood pressure management vs usual care, stratified by the antihypertensive agent administered. Abbreviation: GTN = glyceryl trinitrate.

Prehospital BP-lowering therapies were not associated with good functional outcome (RR: 1.07; 95% CI, 0.52—2.19; *P* = .859; 3 studies; *I*^2^ = 50%; *P* for Cochran *Q* = 0.14; Figure 2) and lower risk of all-cause mortality (RR: 0.90; 95% CI, 0.60—1.35; *P* = .615; 4 studies; *I*^2^ = 68%; *P* for Cochran *Q* = 0.02; Figure 3) at 3 months, compared to those receiving usual care. Notably, subgroup analyses revealed non-significant subgroup differences between the different antihypertensive agents used, for the primary efficacy (*P* for subgroup differences = 0.06; Figure 2) and safety (*P* for subgroup differences = 0.65; Figure 3) outcomes.

**Figure 4:**
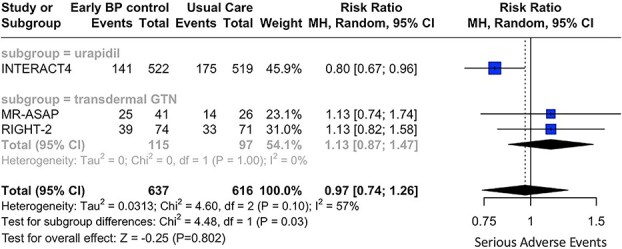
Forest plot presenting the risk ratio of serious adverse events at 3 months among patients with acute hemorrhagic stroke receiving prehospital blood pressure management vs usual care, stratified by the antihypertensive agent administered. Abbreviation: GTN = glyceryl trinitrate.

**Figure 5:**
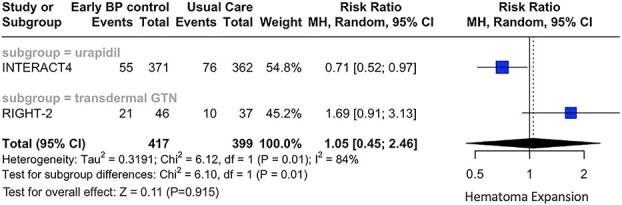
Forest plot presenting the risk ratio of hematoma expansion at 3 months among patients with acute hemorrhagic stroke receiving prehospital blood pressure management vs usual care, stratified by the antihypertensive agent administered. Abbreviation: GTN = glyceryl trinitrate.

Concerning secondary outcomes, similar rates of SAE (RR: 0.97; 95% CI, 0.74—1.26; *P* = .802; 3 studies; *I*^2^ = 57%; p for Cochran *Q* = 0.10; Figure 4) and HE (RR: 1.05; 95% CI, 0.45—2.46; *P* = .915; 2 studies; *I*^2^ = 84%; *P* for Cochran Q = 0.01; Figure 5) were documented in the two treatment groups. Subgroup analyses revealed significant differences between the antihypertensive agents used, with regard to both secondary outcomes [(*P* for subgroup differences with respect to SAE: 0.03; Figure 4) and (*P* for subgroup differences with respect to HE: 0.01; Figure 5)]. More specifically, urapidil was associated with lower risk of SAE (RR: 0.80; 95% CI, 0.67—0.96; 1 study; Figure 4) and HE (RR: 0.71; 95% CI, 0.52—0.97; 1 study; Figure 5). In contrast, transdermal glyceryl trinitrate was associated with numerically higher rates of SAE (RR: 1.13; 95% CI, 0.87—1.47; 2 studies; Figure 4) and HE (RR: 1.69; 95% CI, 0.91—3.13; 1 study; Figure 5).

## Discussion

In this systematic review and meta-analysis of four available RCTs, comprising 1259 patients with acute hemorrhagic stroke and elevated BP, we documented that prehospital BP management did not improve 3-month functional outcome and did not reduce 3-month mortality, HE and SAE. Nevertheless, numerically lower rates of mortality and higher rates of good functional outcome were documented among patients treated with BP-lowering therapies in the prehospital setting. Significant subgroup differences were detected with regard to the antihypertensive agents used, with the α-adrenoreceptor blocker urapidil showing a safer profile regarding SAEs and HE. To the best of our knowledge, this is the first systematic review and meta-analysis investigating this specific research question.

**Table 1:** Basic characteristics of studies included in the systematic review and meta-analysis

**First author, study name**	**Year**	**Study design**	**Data collection interval**	**Diagnosis**	**Initiation of prehospital intervention**	**Total patients receiving early BP control, n**	**Antihypertensive agent**	**Male, *N*, %**	**Mean age, years (SD)**	**Total patients receiving usual care, n**	**Usual care**	**Male, *N*, %**	**Mean age, years (SD)**
Li et al. INTERACT4^10^	2024	Phase 3—open-label, randomized trial, with blinded outcome assessment	03.2020—08.2023	Hemorrhagic stroke, including both intracerebral haemorrhage and a small group (12 patients) with subarachnoid haemorrhage	Within 2 h after the onset of symptoms or last known well	**522**	Intravenous α-adrenoreceptor blocker, urapidil	NA	NA	519	Usual blood-pressure management (ie, commencement of blood-pressure management on arrival at the hospital)	NA	NA
											For patients assigned to usual care, treatment to lower BP was used in the ambulance only for systolic BP of 220 mmHg or higher or for diastolic BP of 110 mmHg or higher		

Van den Berg et al. MR-ASAP^9^	2022	Phase 3—investigator-initiated, randomized, open-label, blinded endpoint trial	04.2018—02.2021	Intracerebral haemorrhage	Within 3 h of symptom onset	**41**	Transdermal glyceryl trinitrate	NA	NA	26	Patients in the control group received standard care, which consisted of admission to the acute stroke unit when a diagnosis of stroke was made	NA	NA
	
Bath et al. RIGHT-2^8^	2019	Single blind blinded endpoint—Iia (paramedic-delivered, ambulance-based, single-city prospective single-blind randomized controlled trial with blinded outcome assessment)	10.2015—05.2018	Intracerebral haemorrhage	Patients were recruited within 4 h of symptom onset. The first treatment (glyceryl trinitrate or sham) was administered immediately after randomization in the ambulance	**74**	Transdermal glyceryl trinitrate	35 (47.3)	73.5 (12)	71	Sham (DuoDERM hydrocolloid dressing; Convatec, Flintshire United Kingdom)	29 (40.8)	72.7 (14)

Ankolekar et al. RIGHT^7^	2013	Multicenter, randomized, double-blind, placebo-controlled, pivotal clinical trial	02.2010—12.2011	Intracerebral haemorrhage	Patients were recruited within 4 h of symptom onset. The first treatment (GTN or sham) was administered immediately after randomization in the ambulance	**5**	Transdermal glyceryl trinitrate	NA	NA	1	None	NA	NA
											Patients randomized to the control group had a gauze dressing applied in a similar position to provide blinding of treatment to the patient		

Abbreviations: GTN = glyceryl trinitrate; NA = not available.

Prehospital management of BP in acute hemorrhagic stroke might be a game changer and theoretically lead to better functional outcomes. The challenge of selecting the right patient for the right treatment is an ongoing research and development topic, and the data from the 4 RCTs included in this meta-analysis with different designs, including mixed stroke populations (patients with acute ischemic stroke, acute hemorrhagic stroke and stroke mimics) really resembles the true nature of the prehospital environment.

The optimal in-hospital strategy regarding the BP management among patients with acute hemorrhagic stroke is still not firmly established. However, BP reduction has been shown to reduce HE in different meta-analyses of available RCTs, assessing the BP management in the acute hemorrhagic setting.^18^^,^^19^ INTERACT2 and ATACH-II trials have provided such evidence in acute ICH patients treated in the hospital setting.^6^^,^^20^^,^^21^

Interestingly, INTERACT-4 was the first to date RCT indicating divergent effects of prehospital BP management among patients with acute ischemic (harmful effect) and hemorrhagic (beneficial effect) stroke.^10^ Nevertheless, specific parameters, such as BP variability, have not been evaluated in the RCTs included in the present meta-analysis.^22^ The sole evidence available regarding the value of BP variability in the prehospital setting originates from the FAST-MAG trial, revealing a negative association between elevated BP variability and early functional outcomes.^23^

Uncertainty also remains about the most effective and safe agent for lowering BP after acute ICH in the prehospital setting. Our meta-analysis included RCTs investigating two different BP-lowering strategies at prehospital settings that were administered via intravenous or transdermal routes. Our prespecified subgroup analysis revealed that the effects of these two interventions varied, with patients treated with the fast-acting, intravenous α-adrenoreceptor blocker urapidil appearing to have a trend towards a better functional outcome and lower rates of mortality, and significantly lower rates of HE and adverse events compared to those treated with transdermal glyceryl trinitrate. Previous European Stroke Organisation guidelines on BP management in acute ischemic or hemorrhagic stroke had already recommended against the use of glyceryl trinitrate due to potential harm attributed to their deleterious effect of vasodilation.^6^ Glyceryl trinitrate has a much longer half-life compared to urapidil and may adversely affect early haemostasis by opposing vasoconstriction and suppressing platelet aggregation, potentially resulting in increased hematoma volume.^19^^,^^24^

Notably, recent European Stroke Organisation guidelines provided a recommendation on using MSUs for the prehospital management of suspected stroke.^25^ Especially for the patients with ICH, the MSU management was associated with a higher proportion of patients being primarily transported to tertiary care stroke centres.^25^ Moreover, considering that MSUs are emergency ambulances equipped with a computed tomography, individually-adjusted prehospital BP lowering in this setting may enhance its efficacy in ICH patients.^26–30^

**Table 2:** Overview of analyses for the outcomes of interest

**Outcome**	**Effect measure (RR)**		**Value (95% CI)**	***P*-value**	**No. of studies**	***I*** ^**2**^ **(*P* for Cochrane *Q*)**	**Subgroup differences for different BP-lowering strategies**
Primary efficacy outcome							
Good functional outcome	Total	1.07	(0.52–2.19)	.859	3	50% (0.14)	0.06
	Subgroup = urapidil	1.45	(1.17– 1.79)	<.001	1	–	
	Subgroup = transdermal GTN	0.61	(0.26—1.47)	.492	2	0% (0.49)	
Primary safety outcome							
All-cause mortality	Total	0.90	(0.60– 1.35)	.615	4	68% (0.02)	0.65
	Subgroup = urapidil	0.79	(0.65– 0.96)	.021	1	–	
	Subgroup = transdermal GTN	0.92	(0.48– 1.76)	.805	3	69% (0.04)	
Secondary safety outcomes							
SAE	Total	0.97	(0.74–1.26)	.802	3	57% (0.10)	0.03
	Subgroup = urapidil	0.80	(0.67– 0.96)	.019	1	–	
	Subgroup = transdermal GTN	1.13	(0.87–1.47)	.349	2	0% (1.00)	
Hematoma expansion	Total	1.05	(0.45–2.46)	.915	2	84% (0.01)	0.01
	Subgroup = urapidil	0.71	(0.52–0.97)	.031	1	–	
	Subgroup = transdermal GTN	1.69	(0.91–3.13)	.095	1	–	

Abbreviations: BP = blood pressure; GTN = glyceryl trinitrate; RR = risk ratio; SAE = serious adverse event.

Notwithstanding its strengths, this meta-analysis has several methodological nuances. First, the overall number of included eligible studies in this meta-analysis was small (*n* = 4) limiting the statistical robustness of the reported associations. Second, significant heterogeneity was detected in the analysis of all outcomes, probably due to the substantial differences in inclusion criteria, randomization processes and antihypertensive agents used. Third, the open label design of the majority of included studies and the data availability only from trials enrolling acute ischemic stroke patients, acute hemorrhagic stroke patients and stroke mimics represents an additional crucial methodological shortcoming. Fourth, due to limited or unavailable data, we were unable to adjust for potential confounders, including the speed and the degree of BP lowering, and the probable underlying ICH aetiology. Additional subgroup analyses to identify patient populations potentially responsive to prehospital BP management were also not feasible due to limited available data. Moreover, a lack of evidence regarding high systolic BP variability in the hyperacute phase of hemorrhagic stroke did not allow further evaluation of its possible effect on functional outcome. Finally, the small sample sizes of many studies in the current meta-analysis may lead to an overestimation of their weight on the pooled results. Conducting an Individual Patient Data Meta-Analysis (IPDMA) could provide a means to mitigate these limitations.

In summary, our meta-analysis indicates that prehospital BP-lowering in acute hemorrhagic stroke does not improve functional outcome and survival. Future RCTs conducted in MSUs, and exclusively focusing on patients with acute hemorrhagic stroke, are required to provide definitive data and answer this intriguing research question.

## Supplementary Material

aakaf023_Revised_SUPPLEMENTAL_MATERIAL

## Data Availability

All data needed to evaluate the conclusions in the paper are present in the main manuscript and in the supplemental material. Additional data related to this paper may be requested from the corresponding author, upon reasonable request.

## References

[1] Qureshi AI . The importance of acute hypertensive response in ICH. Stroke. 2013;44:S67-S69. 10.1161/STROKEAHA.111.000758 23709735

[2] Owens WB . Blood pressure control in acute cerebrovascular disease. J Clin Hypertens (Greenwich). 2011;13:205-211. 10.1111/j.1751-7176.2010.00394.x 21366852 PMC8673094

[3] Vemmos KN, Tsivgoulis G, Spengos K, et al. U-shaped relationship between mortality and admission blood pressure in patients with acute stroke. J Intern Med. 2004;255:257-265. 10.1046/j.1365-2796.2003.01291.x 14746563

[4] Chang JJ, Khorchid Y, Dillard K, et al. Elevated pulse pressure levels are associated with increased In-hospital mortality in acute spontaneous intracerebral hemorrhage. Am J Hypertens. 2017;30:719-727. 10.1093/ajh/hpx025 28430838

[5] Steiner T, Purrucker JC, Aguiar de Sousa D, et al. European Stroke Organisation (ESO) and European Association of Neurosurgical Societies (EANS) guideline on stroke due to spontaneous intracerebral haemorrhage. Eur Stroke J. 2025;10:1007-1086. 10.1177/23969873251340815 PMC12098356 40401775

[6] Sandset EC, Anderson CS, Bath PM, et al. European Stroke Organisation (ESO) guidelines on blood pressure management in acute ischaemic stroke and intracerebral haemorrhage. Eur Stroke J. 2021;6:XLVIII-LXXXIX. 10.1177/23969873211012133. PMC8370078 34780578

[7] Ankolekar S, Fuller M, Cross I, et al. Feasibility of an ambulance-based stroke trial, and safety of glyceryl trinitrate in ultra-acute stroke: the rapid intervention with glyceryl trinitrate in hypertensive stroke trial (RIGHT, ISRCTN66434824). Stroke. 2013;44:3120-3128. 10.1161/STROKEAHA.113.001301 24003041

[8] Investigators R-. Prehospital transdermal glyceryl trinitrate in patients with ultra-acute presumed stroke (RIGHT-2): an ambulance-based, randomised, sham-controlled, blinded, phase 3 trial. Lancet. 2019;393:1009-1020. 10.1016/S0140-6736(19)30194-1 30738649 PMC6497986

[9] van den Berg SA, Uniken Venema SM, Reinink H, et al. Prehospital transdermal glyceryl trinitrate in patients with presumed acute stroke (MR ASAP): an ambulance-based, multicentre, randomised, open-label, blinded endpoint, phase 3 trial. Lancet Neurol. 2022;21:971-981. 10.1016/S1474-4422(22)00333-7 36058230

[10] Li G, Lin Y, Yang J, et al. Intensive ambulance-delivered blood-pressure reduction in hyperacute stroke. N Engl J Med. 2024;390:1862-1872. 10.1056/NEJMoa2314741 38752650

[11] Liu Y, Tan Y, Wan J, et al. Prehospital blood pressure lowering in patients with ultra-acute presumed stroke: a systematic review and meta-analysis. PloS One. 2025;20:e0326494. 10.1371/journal.pone.0326494 40668787 PMC12266422

[12] Page MJ, McKenzie JE, Bossuyt PM, et al. Updating guidance for reporting systematic reviews: development of the PRISMA 2020 statement. J Clin Epidemiol. 2021;134:103-112. 10.1016/j.jclinepi.2021.02.003 33577987

[13] Richardson WS, Wilson MC, Nishikawa J, Hayward RS. The well-built clinical question: a key to evidence-based decisions. ACP J Club. 1995;123:A12-A13. 10.7326/ACPJC-1995-123-3-A12 7582737

[14] Sterne JAC, Savović J, Page MJ, et al. RoB 2: a revised tool for assessing risk of bias in randomised trials. BMJ. 2019;366:l4898. 10.1136/bmj.l4898 31462531

[15] Balduzzi S, Rücker G, Schwarzer G. How to perform a meta-analysis with R: a practical tutorial. Evid Based Ment Health. 2019;22:153-160. 10.1136/ebmental-2019-300117 31563865 PMC10231495

[16] DerSimonian R, Laird N. Meta-analysis in clinical trials revisited. Contemp Clin Trials. 2015;45:139-145. 10.1016/j.cct.2015.09.002 26343745 PMC4639420

[17] Egger M, Davey Smith G, Schneider M, et al. Bias in meta-analysis detected by a simple, graphical test. BMJ. 1997;315:629-634. 10.1136/bmj.315.7109.629 9310563 PMC2127453

[18] Tsivgoulis G, Katsanos AH. Intensive blood pressure reduction in acute intracerebral hemorrhage: a meta-analysis. Neurology. 2015;84:2464-2465. 10.1212/WNL.0000000000001696 26078404

[19] Moullaali TJ, Wang X, Sandset EC, et al. Early lowering of blood pressure after acute intracerebral haemorrhage: a systematic review and meta-analysis of individual patient data. J Neurol Neurosurg Psychiatry. 2022;93:6-13. 10.1136/jnnp-2021-327195 34732465 PMC8685661

[20] Anderson CS, Heeley E, Huang Y, et al. Rapid blood-pressure lowering in patients with acute intracerebral hemorrhage. N Engl J Med. 2013;368:2355-2365. 10.1056/NEJMoa1214609 23713578

[21] Qureshi AI, Palesch YY, Barsan WG, et al. Intensive blood-pressure lowering in patients with acute cerebral hemorrhage. N Engl J Med. 2016;375:1033-1043. 10.1056/NEJMoa1603460 27276234 PMC5345109

[22] Zompola C, Palaiodimou L, Voumvourakis K, et al. Blood pressure variability in acute stroke: a narrative review. J Clin Med. 2024;13:20240329. 10.3390/jcm13071981 PMC11012361 38610746

[23] Oh DM, Shkirkova K, Poblete RA, et al. Association between hyperacute blood pressure variability and hematoma expansion after intracerebral hemorrhage: secondary analysis of the FAST-MAG database. Neurocrit Care. 2023;38:356-364. 10.1007/s12028-022-01657-2 36471183

[24] Li Q, Warren AD, Qureshi AI, et al. Ultra-early blood pressure reduction attenuates hematoma growth and improves outcome in intracerebral hemorrhage. Ann Neurol. 2020;88:388-395. 10.1002/ana.25793 32453453 PMC8697414

[25] Walter S, Audebert HJ, Katsanos AH, et al. European Stroke Organisation (ESO) guidelines on mobile stroke units for prehospital stroke management. Eur Stroke J. 2022;7:XXVII, LIX. 20220209. 10.1177/23969873221079413 35300251 PMC8921783

[26] Ibsen J, Hov MR, Tokerud GE, et al. Prehospital computed tomography in a rural district for rapid diagnosis and treatment of stroke. Eur Stroke J. 2025;10:84-91. 10.1177/23969873241267084 39340436 PMC11556544

[27] Schwabauer E, Piccininni M, Freitag E, et al. Effects of mobile stroke unit dispatch on blood pressure management and outcomes in patients with intracerebral haematoma: results from the Berlin_Prehospital or usual care delivery in acute stroke (B_PROUD) controlled intervention study. Eur Stroke J. 2024;9:366-375. 10.1177/23969873231213156 38014623 PMC11318420

[28] Bowry R, Parker SA, Bratina P, et al. Hemorrhage enlargement is more frequent in the first 2 hours: a prehospital mobile stroke unit study. Stroke. 2022;53:2352-2360. 10.1161/STROKEAHA.121.037591 35369716

[29] Fassbender K, Merzou F, Lesmeister M, et al. Impact of mobile stroke units. J Neurol Neurosurg Psychiatry. 2021;92:815-822. 10.1136/jnnp-2020-324005 34035130 PMC8292607

[30] Cooley SR, Zhao H, Campbell BCV, et al. Mobile stroke units facilitate prehospital management of intracerebral hemorrhage. Stroke. 2021;52:3163-3166. 10.1161/STROKEAHA.121.034592 34187178

