# Supplementary material for: Prehospital blood pressure lowering in acute hemorrhagic stroke: a systematic review and meta-analysis of randomized controlled clinical trials
Source: Eur Stroke J. 2026 Jan 1;11(1):aakaf023. doi: 10.1093/esj/aakaf023 (PMC12866655; doi:10.1093/esj/aakaf023)
Supplement: aakaf023_Revised_SUPPLEMENTAL_MATERIAL [file aakaf023_revised_supplemental_material.docx]

**SUPPLEMENTAL MATERIAL**

**TITLE:** Prehospital blood pressure lowering in acute hemorrhagic stroke: A systematic review and meta-analysis of randomized-controlled clinical trials

**Complete search algorithm used in PUBMED search**

**Complete search algorithm used in SCOPUS search**

**Supplementary Tables:**

- **Supplementary Table-S1:** Excluded Studies with Reasons for Exclusion

**Supplementary Figures:**

- **Supplementary Figures-S1 & S2:** Risk of bias summary of included randomized clinical trials.

**References used in the Supplement.**

**Complete search algorithm used in PUBMED search**

("prehospital"[All Fields] OR "prehospitally"[All Fields]) AND ("stroke"[MeSH Terms] OR "stroke"[All Fields] OR "strokes"[All Fields] OR "stroke s"[All Fields]) AND ("blood pressure"[MeSH Terms] OR ("blood"[All Fields] AND "pressure"[All Fields]) OR "blood pressure"[All Fields] OR "blood pressure determination"[MeSH Terms] OR ("blood"[All Fields] AND "pressure"[All Fields] AND "determination"[All Fields]) OR "blood pressure determination"[All Fields] OR "arterial pressure"[MeSH Terms] OR ("arterial"[All Fields] AND "pressure"[All Fields]) OR "arterial pressure"[All Fields])

**Complete search algorithm used in SCOPUS search**

(TITLE-ABS-KEY (prehospital) AND TITLE-ABS-KEY (stroke) AND TITLE-ABS-KEY (blood AND pressure))

**SUPPLEMENTAL TABLES**

**Supplemental Table S1:** Excluded Studies with Reasons for Exclusion

|  | **Reason for Exclusion** |
| --- | --- |
| **Shaw et al, PIL-FAST^1^** | No data on blood pressure management among acute hemorrhagic stroke patients in the prehospital setting. |
| **Saver et al, FAST-MAG^2^** | No data on blood pressure management among acute hemorrhagic stroke patients in the prehospital setting. |
| **The ENOS Trial Investigators, ENOS^3^** | No data on blood pressure management among acute hemorrhagic stroke patients in the prehospital setting. |
| **Wang et al^4^** | Systematic review and meta-analysis. |
| **Ibrahim et al^5^** | Systematic review and meta-analysis. |
| **Moullaali et al^6^** | Systematic review and meta-analysis. |

**SUPPLEMENTAL FIGURES**

**Figure S1.:** Traffic Light Plot presenting the quality assessment of included randomized clinical trials using the Cochrane Collaboration tool (RoB V.2).^7^

^
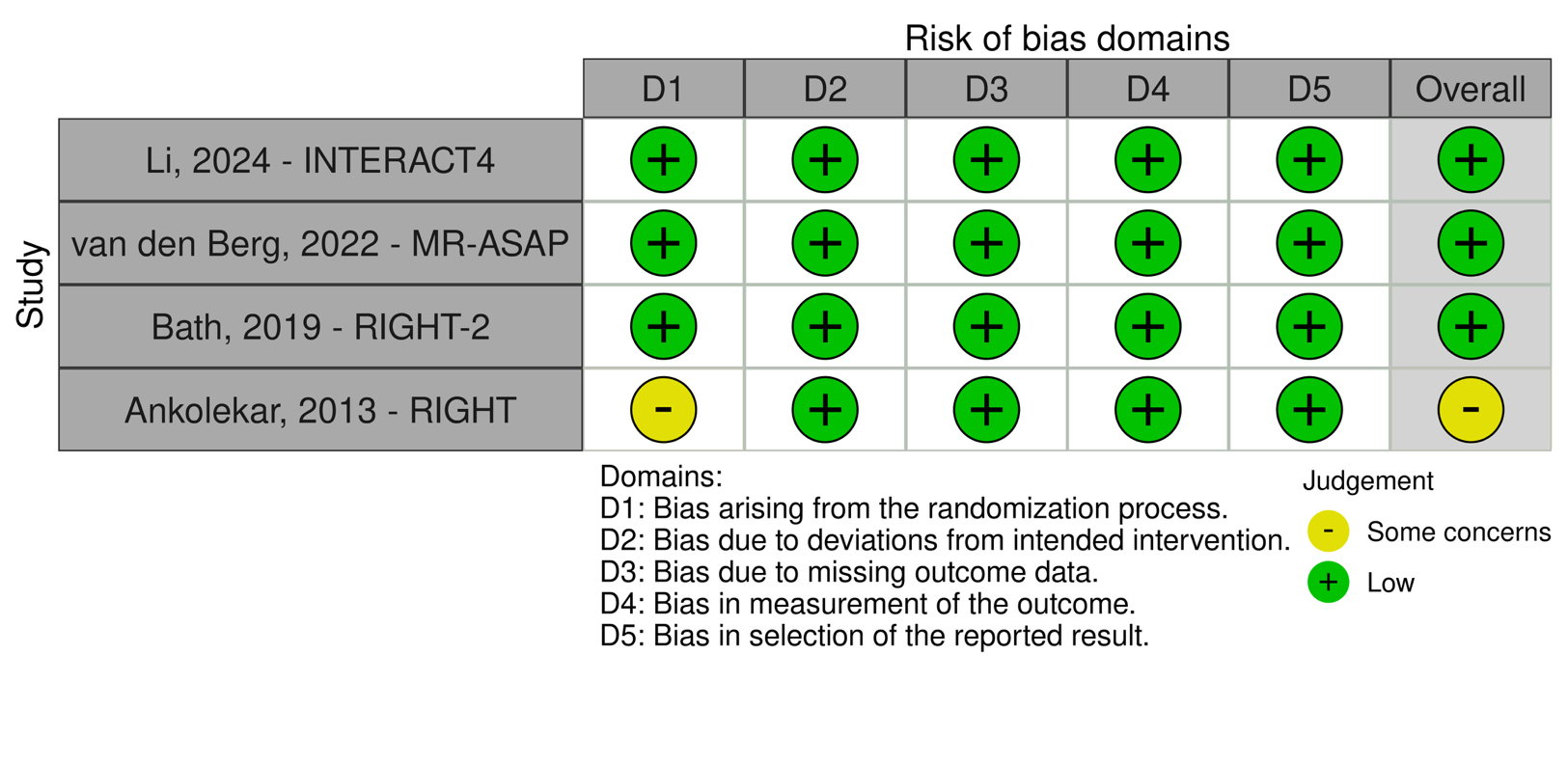
^

**Figure S2.:** Traffic Light Plot presenting the quality assessment of included randomized clinical trials using the Cochrane Collaboration tool (RoB V.2).^7^

^
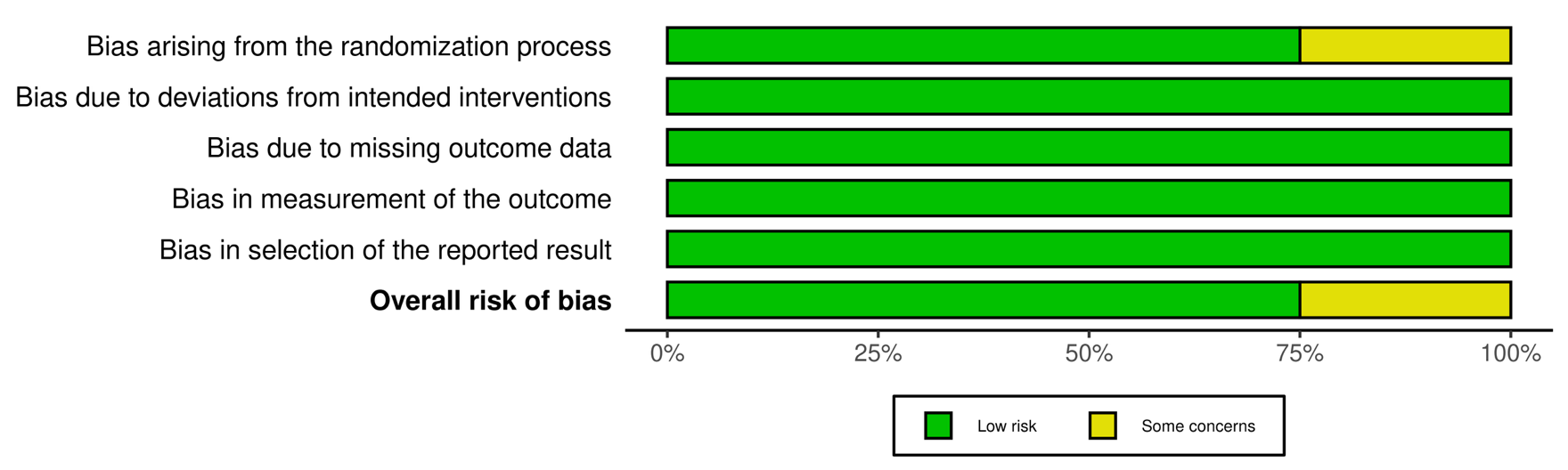
^

**References used in the Supplement.**

1. Shaw L, Price C, McLure S, et al. Paramedic Initiated Lisinopril For Acute Stroke Treatment (PIL-FAST): results from the pilot randomised controlled trial. *Emerg Med J* 2014; 31: 994-999. 20130927. DOI: 10.1136/emermed-2013-202536.

2. Saver JL, Starkman S, Eckstein M, et al. Prehospital use of magnesium sulfate as neuroprotection in acute stroke. *N Engl J Med* 2015; 372: 528-536. DOI: 10.1056/NEJMoa1408827.

3. Investigators ET. Efficacy of nitric oxide, with or without continuing antihypertensive treatment, for management of high blood pressure in acute stroke (ENOS): a partial-factorial randomised controlled trial. *Lancet* 2015; 385: 617-628. 20141021. DOI: 10.1016/S0140-6736(14)61121-1.

4. Wang X, Yang J, Moullaali TJ, et al. Influence of Time to Achieve Target Systolic Blood Pressure on Outcome After Intracerebral Hemorrhage: The Blood Pressure in Acute Stroke Collaboration. *Stroke* 2024; 55: 849-855. 20240227. DOI: 10.1161/STROKEAHA.123.044358.

5. Ibrahim AA, Khlidj Y, Amin AM, et al. Pre-hospital blood pressure lowering in presumed hyperacute stroke: A systematic review and meta-analysis of randomized controlled trials. *J Stroke Cerebrovasc Dis* 2025; 34: 108158. 20241128. DOI: 10.1016/j.jstrokecerebrovasdis.2024.108158.

6. Moullaali TJ, Wang X, Sandset EC, et al. Early lowering of blood pressure after acute intracerebral haemorrhage: a systematic review and meta-analysis of individual patient data. *J Neurol Neurosurg Psychiatry* 2022; 93: 6-13. 20211103. DOI: 10.1136/jnnp-2021-327195.

7. Sterne JAC, Savović J, Page MJ, et al. RoB 2: a revised tool for assessing risk of bias in randomised trials. *BMJ* 2019; 366: l4898. 20190828. DOI: 10.1136/bmj.l4898.
